# Authors’ Reply: Methodological Considerations for a Diabetes Family-Based eHealth Intervention

**DOI:** 10.2196/52204

**Published:** 2023-09-18

**Authors:** Yuheng Feng, Xiaohong Li

**Affiliations:** 1 Department of Health Policy and Management School of Public Health Fudan University Shanghai China

**Keywords:** public health, type 2 diabetes mellitus, intervention, randomized controlled trial, community health center

We greatly appreciate the authors’ interest and comments [1] on our eHealth family-based intervention program [2]. We hope that our responses are beneficial to the article’s readership.

## Intervention Program Development

It is a common phenomenon that patients with type 2 diabetes mellitus (T2DM) have poor glucose control [3]. Clinical inertia, including physician related, health care system related, and patient related, is the main reason [4]. Our study developed an eHealth family-based intervention program, which decreased patient-related inertia. We interviewed endocrinologists and community physicians and found that physician and health care inertia existed. Some patients with T2DM with very poor glucose control failed to visit tertiary or secondary hospitals to adjust their medication.

## Changes in Self-Care Activities

Changes in self-care activities were not substantial. There are two possible reasons. First, most patients with a long disease course had baseline self-care activities that were better than patients with a short disease course. So it was difficult to substantially enhance self-care activities. Second, the intervention intensity was not very high because we aimed to develop a generalizable labor-saving intervention model. Although differences were not substantial, it indicates that the eHealth family-based intervention program is effective.

The general diet and blood sugar test had the most significant effect on changes in hemoglobin A_1c_ (HbA_1c_). During the intervention implementation, family members mainly concentrated on how to help patients with T2DM maintain a healthy diet. Regarding glucose self-monitoring, family members can help them test regularly for the glucose status and prompt them to follow the physician’s suggestion.

## Frequency of Doctor Visits in Control Group

According to the *National Standard for Basic Public Health Services*, community physicians need to follow up with patients registered in the community system once every 3 months. Our patients were recruited from this system. The frequency of doctor visits in the control group was the same as in the intervention group.

## Monitoring of Treatment Changes

Due to workforce deficiencies in community health centers caused by the COVID-19 outbreak, we did not adjust oral medication or insulin. However, treatment changes should be monitored carefully. More detailed information could better explain how the intervention influenced the patients’ glucose control.

## Subanalysis of HbA1c Changes in Correlation With Their Baseline Levels

We considered baseline HbA_1c_ levels as a covariable. The covariance analysis indicated that patients with T2DM with worse baseline HbA_1c_ levels get better intervention effectiveness, similar to Brož et al’s [5] study, which indicates that priorities should be given to patients with T2DM and poorer glucose control.

## Future Studies

To reduce the physician-based and health care–based inertia, measures targeted toward the health care system and physicians should be implemented. Future studies could be focused on physician-based and health care–based factors. More attention could be paid to the mechanism of improving the referral system and community physicians’ skills based on the medical alliance modes [6]. The feedback mechanism of two-way referral also should be improved, which will help the community physicians know whether patients with T2DM follow suggestions and provide targeted health education to those who do not follow suggestions.

## Abbreviations

HbA_1c_: hemoglobin A_1c_
T2DM: type 2 diabetes mellitus
